# Modularity and evolution of flower shape: the role of function, development, and spandrels in *Erica*

**DOI:** 10.1111/nph.16337

**Published:** 2020-01-08

**Authors:** Dieter Reich, Andreas Berger, Maria von Balthazar, Marion Chartier, Mahboubeh Sherafati, Jürg Schönenberger, Sara Manafzadeh, Yannick M. Staedler

**Affiliations:** ^1^ Department of Botany and Biodiversity Research Division of Evolutionary and Systematic Botany University of Vienna Rennweg 14 Vienna 1030 Austria; ^2^ Department of Botany and Biodiversity Research Division of Structural and Functional Botany University of Vienna Rennweg 14 Vienna 1030 Austria; ^3^ Department of Plant Biology Faculty of Biological Sciences Tarbiat Modares University Tehran 14115‐154 Iran; ^4^ Department of Environmental Systems Science ETH Zurich Universitätstrasse 16 Zürich 8092 Switzerland

**Keywords:** developmental modularity, flower shape, functional modularity, integration, modularity, pollination syndrome, spandrel

## Abstract

- Flowers have been hypothesized to contain either modules of attraction and reproduction, functional modules (pollination‐effecting parts) or developmental modules (organ‐specific). Do pollination specialization and syndromes influence floral modularity?
- In order to test these hypotheses and answer this question, we focused on the genus *Erica*: we gathered 3D data from flowers of 19 species with diverse syndromes via computed tomography, and for the first time tested the above‐mentioned hypotheses via 3D geometric morphometrics. To provide an evolutionary framework for our results, we tested the evolutionary mode of floral shape, size and integration under the syndromes regime, and – for the first time – reconstructed the high‐dimensional floral shape of their most recent common ancestor.
- We demonstrate that the modularity of the 3D shape of generalist flowers depends on development and that of specialists is linked to function: modules of pollen deposition and receipt in bird syndrome, and access‐restriction to the floral reward in long‐proboscid fly syndrome. Only size and shape principal component 1 showed multiple‐optima selection, suggesting that they were co‐opted during evolution to adapt flowers to novel pollinators. Whole floral shape followed an Ornstein–Uhlenbeck (selection‐driven) evolutionary model, and differentiated relatively late.
- Flower shape modularity thus crucially depends on pollinator specialization and syndrome.

## Introduction

From the bacterial flagellum (McAdams *et al.*, 2004) to the skull shape of dinosaurs (Fabbri *et al.*, 2017), modular organization pervades life's phenotypes (Wagner *et al.*, 2007).

Modules are subsets of traits that tend to vary in a coordinated manner (i.e. they are integrated) and relatively independently from other such subsets (Klingenberg, 2014). Relative independence of modules allows for evolutionary tinkering to take place in one module without much affecting the others (Alon, 2003; Kirsten & Hogeweg, 2011). Modular organization is thus not only a key feature of the structural complexity of life, but also a key feature for its evolvability (Wagner *et al.*, 2007). Despite the fundamentally modular structure of plants (see Ottaviani *et al.*, 2017, and references therein), historically, most studies of modularity have been, and still are, focused on animals (Klingenberg, 2014; Esteve‐Altava 2017) (see Supporting Information Notes S1).

In her seminal work, Raissa Berg hypothesized that the variation of traits in specialized flowers is largely uncorrelated with that of vegetative traits (Berg, 1960), and , thus, that vegetative and reproductive traits form independent modules, which are themselves highly integrated (Wagner & Altenberg, 1996). That flowers are highly integrated organ complexes has become a paradigm among floral biologists (see, e.g. Stebbins, 1950; Faegri & Van Der Pijl, 1966; Stebbins, 1970; Ordano *et al.*, 2008), as is the hypothesis that specialized flowers are more highly integrated than generalist flowers because specialized pollination is expected to drive the evolution of precise, highly coordinated (integrated) floral traits (see, e.g. Berg, 1960; Armbruster *et al.*, 1999; Pérez *et al.*, 2007; Rosas‐Guerrero *et al.*, 2011; Ellis *et al.*, 2014; Gomez *et al.*, 2014, 2016). Evidence has been provided in favour of the latter hypothesis (see, e.g. Meng *et al.*, 2008; Rosas‐Guerrero *et al.*, 2011; Ellis *et al.*, 2014; Gomez *et al.*, 2014) as well as against it (see, e.g. Armbruster *et al.*, 1999; Edwards & Weinig, 2011; Joly *et al.*, 2018). These contrasting results have led to the hypothesis that it is not the total, whole‐flower integration that is subject to pollinator‐mediated selection, but rather its intrafloral structure: its modularity (Ordano *et al.*, 2008; Diggle, 2014; Armbruster & Wege, 2018).

**Figure 1:**
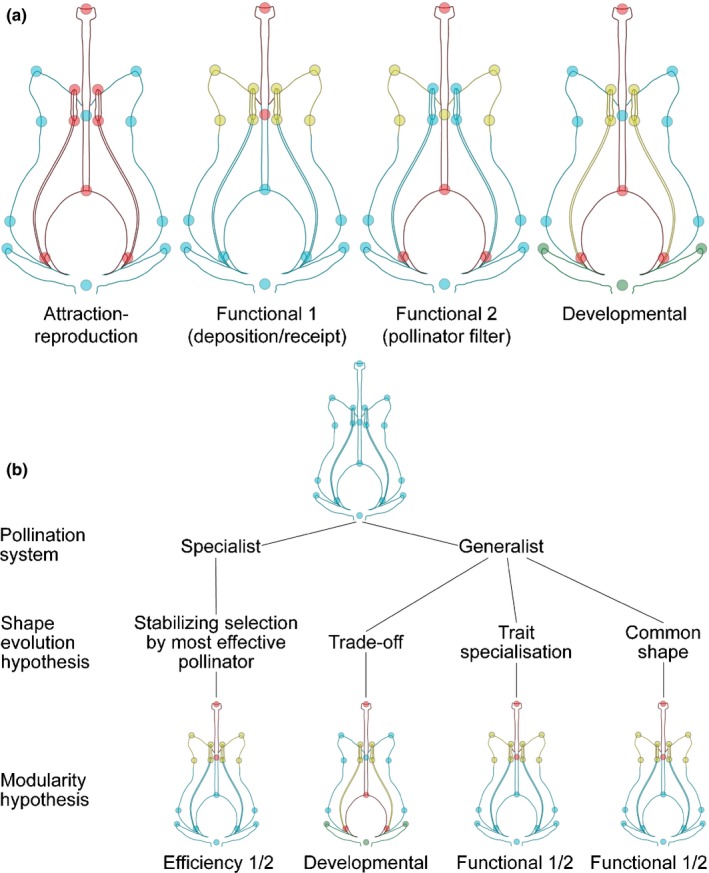
Hypotheses. (a) Modularity hypotheses tested displayed on schematic representation of an *Erica* flower. Left, the *attraction‐reproduction* hypothesis proposes that floral organs group into fertile (stamens and carpel, in red) vs sterile (sepals and petals, in blue) modules. Centre left, the *functional* hypothesis 1 proposes that parts of the flower group in modules directly involved in pollen receipt (joining of the petals and stigma, in red) and deposition (rest of the corolla mouth and stamens, in yellow), and modules that are not (remainder of the flower in blue). Centre right, the *functional* hypothesis 2 proposes that parts of the flower that restrict access to the floral reward (floral neck, in yellow) form a module, that the carpels form a module, and that the rest of the flower also forms a module. Right, the *developmental* hypothesis proposes that parts for the flower group into modules corresponding to their organ identity: sepals (green), petals (blue), stamens (yellow) or carpels (red). (b) Hypotheses graph displaying the tested modularity hypotheses and their relationship to shape evolution hypotheses and pollination system.

Accordingly, the following three explicit hypotheses of intrafloral modularity have been advanced. The first hypothesis is the *attraction‐reproduction modularity hypothesis*; which proposes that flowers are divided into two modules: a module of attraction comprising the petals and the sepals; and a module of reproduction comprising the stamens and the carpels (Esteve‐Altava, 2017) (see Fig. 1a). This hypothesis has been supported in a few studies (Anderson & Busch, 2006; Ashman & Majetic, 2006; Ordano *et al.*, 2008; Tucić *et al.*, 2013; Fornoni *et al.*, 2015). The second hypothesis is the *functional modularity hypothesis*, which proposes that flowers are divided into one or several functional module(s) that comprise: parts from different organs directly effecting reproduction (such as constriction of floral tube, pollen sacs of the stamens and stigmas of the carpels); and a module of attraction (e.g. showy part of petals) (Diggle, 2014). The *functional hypothesis* has been supported in numerous studies (see, e.g. Waitt & Levin, 1993; Conner & Sterling, 1995; Cresswell, 1998; Armbruster *et al.*, 2004; Pérez‐Barrales *et al.*, 2007; Pérez *et al.*, 2007; Bissell & Diggle, 2010; Rosas‐Guerrero *et al.*, 2011; Fornoni *et al.*, 2015; Armbruster & Wege, 2018). *Functional hypotheses* may comprise modules of pollen deposition (on the pollinator), receipt (by the stigma) and the rest of the flower (three modules in total; see functional hypothesis 1 in Fig. 1a) (Diggle, 2014). *Functional hypotheses* also may comprise modules involving putative pollinator filters such as corolla aperture, a female module and the rest of the flower (three modules in total; see functional hypothesis 2 in Fig. 1a). The third hypothesis is the *developmental hypothesis*, according to which floral modularity is dominated by floral developmental factors: thus, each developmental organ class (sepal, petal, stamen and carpel) forms its own, separate module (Conner & Sterling, 1995; Diggle, 2002; Herrera *et al.*, 2002) (four modules in total; see developmental hypothesis in Fig. 1a). This hypothesis is grounded both in developmental genetic data and historical factors. Expression of developmental genes from each organ class are activated by different MADS‐box transcription factor oligomers (Coen & Meyerowitz, 1991; Bartlett, 2017); moreover, the different organs making up a flower have evolved – mostly – from different progenitors, most likely with little functional association for at least *c.* 125 Myr (Morris *et al.*, 2018), from the origin of seed plants to that of flowering plants (Endress, 2001). The *developmental hypothesis* is thus a form of null‐hypothesis for floral modularity (Herrera *et al.*, 2002), which has been supported by a few studies (Waitt & Levin, 1993; Conner & Sterling, 1995; Runions & Geber, 2000; Herrera, 2001; Herrera *et al.*, 2002; Ashman & Majetic, 2006; Armbruster & Wege, 2018).

In flowers with specialized syndromes, due to stabilizing selection by the ‘most effective pollinator’ (Stebbins, 1970; Cresswell, 1998), we expect to observe support for different versions of the *functional modularity* (see Fig. 1b). Most published studies support the *functional modularity* in specialized flower (see, e.g. Conner & Sterling, 1995; Cresswell, 1998; Armbruster *et al.*, 2004; Anderson & Busch, 2006; Pérez‐Barrales *et al.*, 2007; Bissell & Diggle, 2008, 2010; Nattero *et al.*, 2010; Rosas‐Guerrero *et al.*, 2011; Wanderley *et al.*, 2016; Heywood *et al.*, 2017). However, in specialized flowers support also was evidenced for *developmental modularity* (see Waitt & Levin, 1993; Conner & Sterling, 1995, 1997; Conner, 2002; Sánchez‐Lafuente 2002; Pérez‐Barrales *et al.*, 2007; Rosas‐Guerrero *et al.*, 2011; Armbruster & Wege, 2018), as well as for the *attraction‐reproduction hypothesis* (Tucić *et al.*, 2013).

In generalist flowers, three main hypotheses have been advanced to explain how pollinators affect floral shape (see Fig. 1b) (Aigner, 2001; Sahli & Conner, 2011; Joly *et al.*, 2018), each of which implies a different floral modularity. (1) The ‘trade‐off’ hypothesis (Aigner, 2001, 2006; Sahli & Conner, 2011) suggests that a trait change that increases the fitness contribution of one pollinator will decrease the fitness of another. This model predicts that selection by multiple pollinators in multiple directions would cancel each other out, resulting in weak or absent *functional modularity*, in which case *developmental modularity* should be observed instead (Herrera *et al.*, 2002), see Fig. 1(b). (2) The ‘trait specialisation’ hypothesis (Sahli & Conner, 2011) proposes that individual traits are under selection by a subset of pollinators, resulting in flowers that possess different traits adapted to different pollinators, which predicts several, well‐defined *functional modules* (see Fig. 1b). (3) The ‘common shape’ hypothesis (Sahli & Conner, 2011) implies that the different pollinators all select for a common shape, which also predicts the existence of *functional modules* (see Fig. 1b). In generalist species, most studies support the *functional hypothesis* (Conner & Via, 1993; Conner & Sterling, 1995; Conner, 1997; Conner, 2002; Sánchez‐Lafuente 2002; Armbruster *et al.*, 2004; Pérez‐Barrales *et al.*, 2007; Pérez *et al.*, 2007; Rosas‐Guerrero *et al.*, 2011; Fornoni *et al.*, 2015; Armbruster & Wege, 2018); however, the *developmental hypothesis* in flowers also was supported in some studies (Conner & Sterling, 1995; Herrera, 2001; Armbruster & Wege, 2018), as well as the *attraction‐reproduction hypothesis* (Ordano *et al.*, 2008). Therefore, although most studies in both specialized and generalized flowers support the *functional hypotheses*, there is currently no consensus on how pollinator specialization and generalization influence flower modularity.

This lack of consensus could be due to the fact that studies of floral modularity and integration have relied largely on distance‐based morphometrics (but see Gómez *et al.*, 2009, 2014, 2016; Carleial *et al.*, 2017; Joly *et al.*, 2018; Smith & Kriebel, 2018) and have focused mostly on few, unrelated species (but see Pérez *et al.*, 2007; Ordano *et al.*, 2008; Rosas‐Guerrero *et al.*, 2011; Gomez *et al.*, 2014, 2016; Fornoni *et al.*, 2015; Armbruster & Wege, 2018; Joly *et al.*, 2018; Smith & Kriebel, 2018). In the framework of distance‐based morphometrics, 30 points in space (e.g. bases and tips of organs) yield 15 distances (e.g. organ lengths); in the geometric morphometrics (GM) framework, however, the same 30 points encapsulate the information of *all the distances* between *all the 30 points* (i.e. 435 distances). This property makes GM well‐suited to digitizing the complex 3D geometry of flowers and ideally suited for the study of modularity, which focuses on which feature is dependent or independent of which other. The few studies that used GM all were focused on integration and based on 2D pictures. Two‐dimensional pictures not only add noise to the digitization of data (Cardini, 2014), but also are incomplete: in frontal view, pictures miss the organs’ lengths, and in lateral view, the fertile organs (stamens and carpels) are usually largely hidden. Moreover, because previous studies have focused overwhelmingly on few species, macro‐evolutionary aspects of floral integration and modularity are currently understudied. No study has yet addressed if, for a lineage where a number *n* of pollination syndromes (*sensu* Vogel, 1954; Grant & Grant, 1965; Stebbins, 1970; Johnson, 2006) evolved repeatedly, the evolution of floral parameters such as whole flower shape (including reproductive organs), size and integration are affected by pollination syndromes and follow a natural selection model such as an Orstein–Uhlenbeck (OU) process with *n* optima (Beaulieu *et al.*, 2012), or, if these floral parameters are not affected by pollination syndromes, and follow a drift–like model such as the Brownian Motion (BM) (Cavalli‐Sforza & Edwards, 1967) process instead (Harmon *et al.*, 2010).

Answering these questions and testing these hypotheses requires a study system, in which convergent evolution of specialist pollination systems occurred, and that also contains species with generalist pollination; such a system also should possess a constant floral bauplan in order to rigorously homologize structures. *Erica* is such a system: it is a large genus of *c*. 800 species mostly distributed in South Africa (Pirie *et al.*, 2016). Within the many South African members of the genus, evolution of pollination via birds and long‐proboscid flies (LPF) has possibly repeatedly taken place (Pirie *et al.*, 2011), whereas a generalist pollination syndrome has been found to be prevalent in European species (see Table 1). Moreover, the flowers of *Erica* have consistently the same, tetramerous bauplan with mostly eight stamens (Stevens *et al.*, 2004). *Erica* is thus the ideal system to test the effects of pollinator shifts on floral modularity.

**Table 1:** Sampling, pollination syndrome, observed (a, b, e–n) or predicted (in the literature: c, d, or via machine learning: RF, Random Forests), and number of flowers scanned.

Species	Clade^a^	Syndrome	Reference	*n* (flowers)
*Erica australis* L.	Palearctic	gen	b	11
*Erica blandfordia* Andrews	Cape	gen	c, RF	11
*Erica bolusiae* T. M. Salter	Cape	gen	c, RF	10
*Erica brachialis* Salisb.	Cape	bird	c, j, k	14
*Erica capensis* T.M. Salter	Cape	gen	c, n	10
*Erica curviflora* L.	Cape	bird	c, RF	11
*Erica georgica* L. Guthrie & Bolus	Cape	lpf	RF	15
*Erica gracilis* J.C. Wendl.	Cape	gen	l	10
*Erica hirtiflora* Curtis	Cape	gen	c, m	10
*Erica lateralis* Willd.	Cape	gen	c, d, RF	10
*Erica leucotrachela* H.A. Baker	Cape	bird	c, RF	10
*Erica margaritacea* Aiton	Cape	gen	c, RF	13
*Erica melanthera* L.	Cape	gen	c, RF	10
*Erica perspicua* J.C. Wendl.	Cape	bird	e, c, g	10
*Erica scoparia* L.	Palearctic	wind	f	10
*Erica spiculifolia* Salisb.	Palearctic	gen	RF	12
*Erica turgida* Salisb.	Cape	gen	c, RF	12
*Erica vagans* L.	Palearctic	gen	h, i	11
*Erica ventricosa* Thunb.	Cape	lpf	c, d^a^	9

Visitor data from literature, websites and personal observation. Gen, insect generalist pollination syndrome; LPF, long‐proboscid fly. a, (Pirie *et al.*, 2016); b, (Gil‐López *et al.*, 2014); c, (Rebelo *et al.*, 1985); d, (Rebelo *et al.*, 1984); e, (Heystek *et al.*, 2014); f, (Herrera, 1988); g, (Geerts, 2011); h, (Fern & Fern, 2012); i, (Plants_Database, 2019); j, (Turner, 2010); k, (Notten, 2012); l, (Joy Stadler, pers. obs. on cultivated specimen); m, (Arendse, 2015); n, (Cullinan *et al.*, 2019). RF, syndrome predicted via random forests. c and d, contain description of syndromes and attribute different *Erica* species to them.

a Contains mention of observation for this species.

In order to test the above‐mentioned modularity and macro‐evolutionary hypotheses, we generated 3D models of *Erica* flowers, the shape of which we digitized using geometric morphometric landmarks. We then used this shape dataset to test our different modularity hypotheses in *Erica* flowers (attraction‐reproduction, developmental, and functional 1 and 2) with different pollination syndromes (generalist, bird, LPF and wind). We used phylogenetic reconstructions to test if floral parameters (shape, size and integration) evolved under selection driven by pollination syndromes or randomly. We thus aimed to understand: (1) The relative utility of the different components of floral shape (i.e. which Cartesian coordinate of which landmark) and size in predicting pollination syndromes; (2) how floral shape modularity changes with pollination syndromes and floral specialization; (3) the possible evolutionary patterns of floral shape in *Erica*; and (4) which of the natural selection models (i.e. Ornstein–Uhlenbeck) and drift‐like models (i.e. Brownian motion) best explain the evolution of floral shape, size and integration with respect to pollination syndromes.

## Materials and Methods

### Plant material

We analyzed *c*. 10 flowers each from a single genotype representing nineteen species of *Erica* from the collections of the Belvedere Garden (Austrian Federal Gardens). We selected one genotype per species because it afforded us the most reproducible sampling of floral variation; moreover, in different systems, most floral trait variance has been shown to lie within individuals (Williams & Conner, 2001), and trait correlation patterns within individuals have been shown to be similar to that among individuals (Ishii & Morinaga, 2005). We selected species based on their diversity in pollination syndrome (generalist, bird, long‐proboscid flies (LPF) and wind; see Table 1) and broadly representative phylogenetic position. Although limited, our taxon sampling contains both older European lineages and species from the more recently diversified and species rich South‐Western Cape Clade, as defined by Pirie *et al.* (2011, 2016) (for details, see Methods S1; Table 1).

### X‐ray tomography

Flowers were contrasted with phosphotungstic acid, mounted in plastic containers and scanned in an XCT‐200 X‐Ray scanner (Zeiss Microscopy) (Staedler *et al.*, 2013). Scanning conditions are summarized in Table S1. Two hundred and nine flowers were scanned and used for landmarking. The raw scanning data were processed with the XMReconstructor package and reconstructed in Dicom format (for details, see Methods S1; Table S1).

### 3D‐landmarking & geometric morphometrics

**Figure 2:**
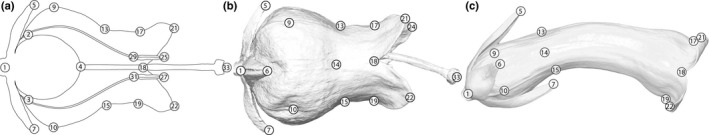
Landmarks. Landmarks used to digitize the shape of *Erica* flowers: (a) on schematic longitudinal section diagram of an *Erica* flower; (b) on a 3D model of an actinomorphic flower (*E. hirtiflora*); and (c) on a 3D model of a zygomorphic flower (*E. leucotrachela*).

Tomography datasets were imported into the Amira v.5.4.1 (Visualisation Sciences Group, SAS) software suite. Geometric morphometric landmarking was carried out on surface models in Amira (generated via the isosurface function). Thirty‐three homologous landmarks were placed on each flower (see Fig. 2a–c; Table S2). Landmark coordinates were exported as .csv files, concatenated and imported in morphoj 1.06d (Klingenberg, 2011). Procrustes fit and calculation of the covariance matrix, Principal Component Analysis (PCA), modularity analyses and allometric regressions were performed in morphoj (see Methods S1 for details).

### Pollination syndrome prediction

We based our categorization of putative pollinators on the work of Rebelo *et al*. (1985), who classified species of *Erica* into pollination syndromes. Pollination syndrome prediction in the Cape Flora often has proven to be accurate (Johnson & Wester, 2017). Additionally, we used published visitor data (available for eight species, see Table 1) and our own observation of *E. gracilis* in cultivation (Supplementary Notes S2) as training data for an artificial intelligence prediction of the visitors of the remaining ten species.

Species with flowers observed to be visited by birds and LPF (see Table 1) were classified into the specialized bird and LPF syndrome. Wind pollination was documented in one species, which was then classified into the wind syndrome. Species with flowers that were observed to be visited by several groups of insects (e.g. bees, hoverflies, beetles, butterflies) that could pollinate the flowers were classified into the generalist syndrome. Using these observations, we identified the floral shape and size components discriminating among pollination types using a Random Forest (RF) classification algorithm (Breiman, 2001). In order to predict pollination syndromes, we used machine learning via the function *randomForest* (randomForest) (Liaw & Wiener, 2002) (see Methods S1 for details).

### Modularity analysis

We used the RV coefficient method of Klingenberg (Klingenberg, 2009), implemented in morphoj (Klingenberg, 2011) to test our modularity hypotheses. The methodology uses the RV coefficient, a multivariate generalization of the squared Pearson coefficient (Escoufier, 1973), as a measure of independence of subsets of the landmark data; given a partition into sets of landmarks, if this partition coincides with the true boundaries between modules, the correlations among sets of landmarks should be minimal (Klingenberg, 2009). We carried out modularity analyses on subsets of our data pooled by syndrome (variation pooled by species). We then calculated the correlation between the shape variation of the sets of landmarks (RV coefficient) of the partitions corresponding to the attraction‐reproduction hypothesis, the developmental hypothesis, and functional hypotheses 1 and 2 (Fig. 1a–d; Table S2) and compared it with that of 100 million random partitions (Klingenberg, 2009) as implemented in morphoj (Klingenberg, 2011). The proportion of partitions with lower RV coefficient than the tested partition (i.e. partitions showing higher among‐set independence) was used as a measure of support for that partition (Young, 2006; Gomez *et al.*, 2014).

### Estimation of size and integration

We measured size as the species‐level average in centroid size, as implemented in morphoj. We calculated integration coefficients at the species level as shape PCA eigenvalue variance scaled by the total variance and number of variables (Klingenberg & Marugan‐Lobon, 2013) as implemented in morphoj (see Table S3).

### Phylogenetic inference

We inferred phylogenetic relationships using DNA sequences from two loci of the chloroplast genome (*trn*LF‐*ndhJ* and *trnT‐L* intergenic spacers) and one loci of the nuclear genome (internal transcribed spacer (ITS)) from 61 pre‐existing sequences of the 19 *Erica* species as ingroup, and *Calluna vulgaris* and *Daboecia cantabrica* as outgroups (see Table S4 for source of the sequences and their GenBank numbers). Divergence time analyses were carried out within a Bayesian framework by employing an uncorrelated lognormal relaxed clock model in Beast v.1.8.4 (Drummond *et al.*, 2012) and applying secondary calibration by using the two previously published nodal ages (Pirie *et al.*, 2016) (see Methods S1 for details).

### Ancestral character state reconstruction

We used a pruned phylogeny (i.e. removing the outgroup) for the 19 *Erica* species included in this study to estimate the probability of the pollination syndrome states for all nodes of the phylogeny. As a demonstration of the potential of this approach, we estimated ancestral states of pollination syndromes using maximum‐likelihood (ML) (Harmon *et al.*, 2010; Revell, 2012) and empirical Bayes (Revell, 2012) methods (for details, see Methods S1; Table S5).

### Models of floral trait evolution: high‐dimensional

We applied a penalized likelihood approach to our high‐dimensional phenotypic dataset of flower shapes of 19 *Erica* species to estimate the fit of three different evolutionary models; Brownian Motion (BM, random walk model), Ornstein–Uhlenbeck (OU, selective peak model) and Early Burst (EB, model of rapid morphological evolution followed by relative stasis) in order to better understand the process of floral‐shape evolution in the clade (Clavel *et al.*, 2018). The analysis was carried out under the *fit_t_pl* function (rpanda) (Morlon *et al.*, 2016), and the best‐fit of the abovementioned three models was assessed using the Generalized Information Criterion (GIC) with the *GIC* function (MVmorph) (Clavel *et al.*, 2015). Finally, we employed the parameters derived from the evolutionary model that best fitted our high‐dimensional data to obtain floral shape reconstructions through time, as implemented in the function *ancestral* and *phyl.pca_pl* (rpanda) (Morlon *et al.*, 2016). To visualize 3D models of the reconstructed ancestral floral shapes at selected nodes, a 3D surface model of a flower of *Erica hirtiflora* (lying approximatively in the middle of the PC1 × PC2 space plot) was warped (distorted) to each target ancestral shape. The warping was carried out by aligning the reconstructed ancestral shape at the selected nodes and the landmark data of the chosen model (*E. hirtiflora*) using a thin plate spline (TPS) interpolation (Wiley *et al.*, 2005), using the function *tps3d* (morphoj) (Schlager, 2017) and the function *extractShape* (Clavel *et al.*, 2018).

### Models of floral trait evolution: unidimensional

In order to understand how changes in pollination syndromes influence the evolution of various continuous unidimensional floral traits of *Erica* (i.e. PC1, PC2, PC3, PC4, PC5, centroid size, and integration), we fitted a series of likelihood models: BM_1_ (random walk model with a single evolutionary rate across all branches) and BM_S_ (random walk model with different rates for each group of taxa based on a single phylogenetic mean); and OU_1_ (selection‐like model with a single rate and a single optimum for all taxa), OU_M_ (selection‐like model with a single rate but different optima for each group of taxa) and OU_MV_ (selection‐like model with different optima and different rates for each group of taxa). The best‐fitting model was determined comparing AICc and AICc weights among the models. All analyses were implemented using R/ouwie (see Methods S1 for details).

## Results

### Pollination syndromes

The floral features used in the RF classification algorithm successfully classified species into pollinator classes. The most important variable for pollinator prediction was tube length (Fig. S1a; Tables S6, S7). The next 15 most important variables were landmarks describing the widest and narrowest positions of the corolla, the ovary/style transition, the meeting point of petal lobes and the position of sepal tips (Fig. S1a; Table S6). For nine of the 10 species for which we predicted pollinators, all flowers were assigned to the same pollination syndrome (Table S8). Flowers of *Erica georgica* were classified either as generalist, bird, LPF or wind syndrome with varying support (Table S8). We assigned *E*. *georgica* to the LPF syndrome because the tube length of all these flowers corresponds to that syndrome (Fig. S1b), and because the shape of the flower and its morphology also corresponds to that syndrome, as defined for *Erica* (Rebelo *et al.*, 1985). Our RF classifications are in agreement with the classification (Rebelo *et al.*, 1985).

### Flower shape PCA

**Figure 3:**
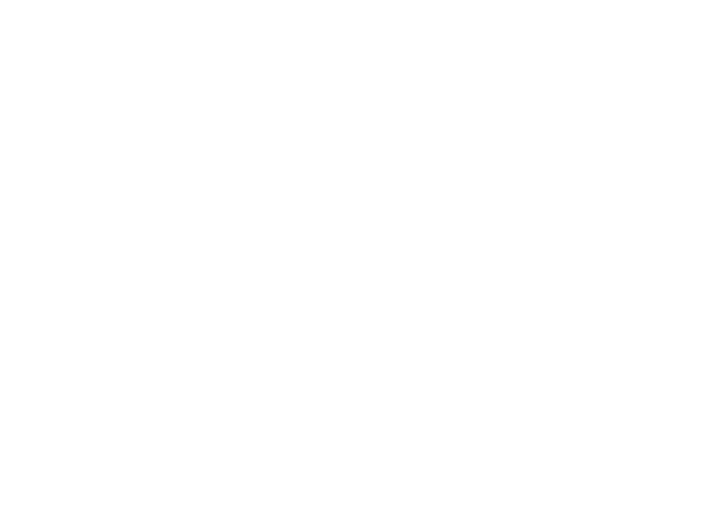
Shape principal component analysis (PCA) and syndromes. Two‐dimensional ordination plot from a PCA analysis of 33 landmarks and 209 individual flowers of 19 *Erica* species. A representative flower surface‐model for each species is plotted next to the dots corresponding to individual flowers of the same species. Colour and shape coding: green‐blue circles, generalist syndrome; orange‐red squares, bird syndrome; pink and purple triangles, long‐proboscid fly syndrome; grey crosses, wind syndrome. Closed symbols: observed visitors, open symbols: predicted visitors. Loadings of axes: *x*‐axis PC1: 38.9% of shape variation, *y*‐axis PC2: 22.1% of shape variation. In order to illustrate changes in floral shape associated with PC1 and PC2, a flower from the centre of the morphospace (*E. hirtiflora*) was distorted according to PC1 and PC2 and plotted along their respective axes.

Together, principal component (PC) 1 and PC2 account for 62% of total shape variation (38.9% for PC1 and 22.1% for PC2). The main distortion along the PC1 is a constriction, elongation and slight curving of the corolla tube. Flowers along PC2 are differentiated mainly by the proximal to medial position of the inflation of the corolla. This varies from globose‐urceolate to tubular‐urceolate flowers along PC1 and cylindrical to ovoid floral shape along PC2. The PC axis‐related distortion along PC1 and PC2 is visualized by an exemplary shape distortion of a flower of *E. hirtiflora* (Fig. 3). The spreading along the two axes did not reflect the phylogeny in separating clades defined by (Pirie *et al.*, 2016) (but see the *Evolution* section below). The convergent evolution of the bird and LPF syndromes in our dataset displays different patterns: the LPF syndrome flowers are tightly clustered in the morphospace whereas the bird syndrome flowers are in two clusters.

### Flower modularity

**Figure 4:**
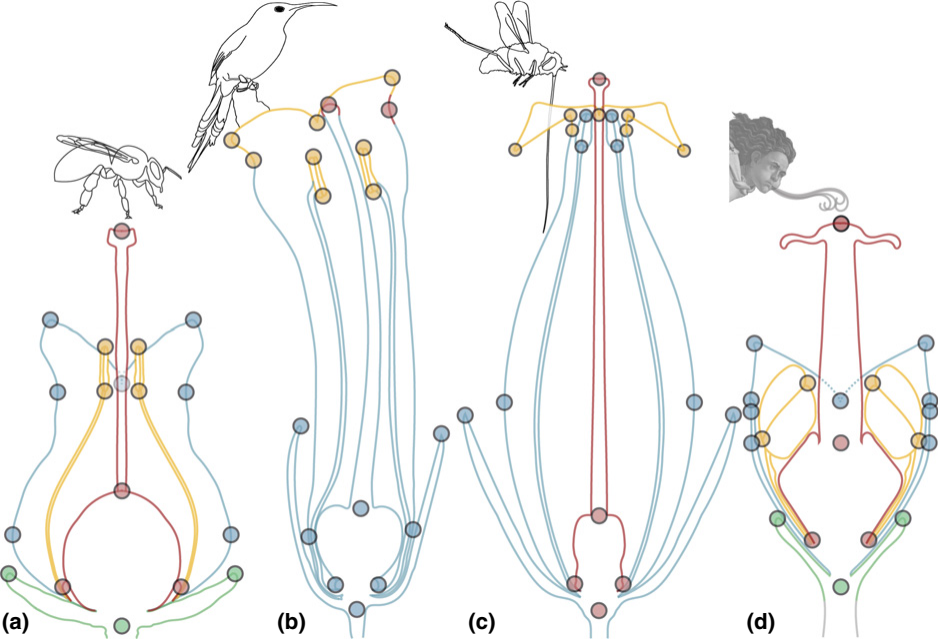
Modules in *Erica* flowers. (a) The best‐supported partition in flowers with generalist syndrome is the *developmental* hypothesis: a 4‐fold partition with each organ class forms one module (each organs class with its own colour). (b) The best‐supported partition in the flowers with bird syndrome is the *functional* hypothesis 1, where the corolla lobes and the stamen form a putative ‘pollen deposition module’ (yellow), and joining of the upper corolla lobes and the stigma form a putative ‘pollen receipt module’ (red). The third set of landmarks comprises the rest of the flower (blue). (c) The best‐supported partition in flowers with long‐proboscid fly syndrome is the *functional* hypothesis 2, where the landmarks on the narrow corolla aperture form a putative ‘restriction module’ (yellow) that restricts access to the floral reward to only insects with very narrow proboscises. A second set of landmarks is formed by the gynoecium (red), and a third set of landmarks comprises the rest of the flower (blue). (d) The best supported partition in flowers with wind syndrome the *developmental* hypothesis: a 4‐fold partition with each organ class forms one module (each organs class with its own colour). Pollinator drawings, originals. Generalists represented by drawing of bee. Character representing the wind: Zephyr from ‘The birth of Venus’ by Sandro Boticelli (*c*. 1480).

In flowers with a generalist syndrome, the best‐supported modularity hypothesis was the *developmental* hypothesis (see Table 2; Figs 4a, S2a–d), although the *functional* hypotheses 1 (pollen deposition and receipt) and 2 (restriction) received weaker support (see Table 2). In flowers with a bird syndrome, the best‐supported modularity hypothesis was the *functional* hypothesis 1 (see Table 2; Figs 4b, S2e–h), although the *developmental* hypothesis received only slightly weaker support (see Table 2). In flowers with a LPF syndrome, the best‐supported modularity hypothesis was the *functional* hypothesis 2 (see Table 2; Figs 4c, S2i–l), whereas *functional* hypothesis 1 received weaker support (see Table 2). In flowers with wind syndrome, the best‐supported modularity hypothesis was the *developmental* hypothesis (see Table 2; Figs 4d, S2m–p). The *attraction‐reproduction* hypothesis was not strongly supported for any pollination syndrome (See Table 2).

**Table 2:** Modularity tests for the attraction‐reproduction, developmental, and functional 1 and 2 hypotheses.

Hypothesis	RV of hypothesis	Lowest RV	Proportion lower RV
Generalist syndrome			
Attraction/reproduction	0.22	0.19	1.40E‐003
**Developmental**	0.12	0.11	**2.30E‐007**
Functional 1	0.13	0.11	4.10E‐005
Functional 2	0.16	0.14	7.33E‐006
Bird syndrome			
Attraction/reproduction	0.4	0.16	3.50E‐002
Developmental	0.19	0.16	3.66E‐006
**Functional 1**	0.15	0.14	**3.02E‐006**
Functional 2	0.29	0.16	3.50E‐003
LPF syndrome			
Attraction/reproduction	0.39	0.3	1.80E‐002
Developmental	0.23	0.17	8.07E‐004
Functional 1	0.17	0.16	4.20E‐006
**Functional 2**	0.23	0.22	**5.50E‐007**
Wind syndrome			
Attraction/reproduction	0.72	0.44	2.50E‐001
**Developmental**	0.43	0.32	**1.70E‐003**
Functional 1	0.47	0.29	4.00E‐002
Functional 2	0.54	0.34	2.60E‐002

Low RV values indicate low correlation, i.e. high independence of the subsets of landmarks (Klingenberg, 2009). The RV values of the partition corresponding to the different hypotheses are compared with that of 100 million random partitions. The lower the proportion of random partitions with better support (with lower RV value) than the hypothesis, the better the support for said hypothesis (most significant values in bold). LPF, long‐proboscid flies.

### Flower allometry

The symmetric component of the entire dataset exhibited significant but weak allometry: 1.17% (*P* = 0.001; see Fig. S3a). If the species are split by pollination syndrome, the proportion of variation explained by allometry (pooled by species) differs according to syndrome (see Notes S2). For the sake of brevity, only the allometric deformation in syndromes for which it is both strong (> 10% predicted shape) and significant (*P* < 0.05) will be detailed here (i.e. LPF and wind syndromes). In the flowers with LPF syndrome, large flowers tend to have a more flask‐shaped corolla, and the landmarks on the mouth of the corolla are closer to the floral axis (Fig. S3b). In the flowers with wind syndrome, large flowers tend to have corolla lobes more open and stamens more exerted (Fig. S3c).

### Ancestral character states reconstruction

**Figure 5:**
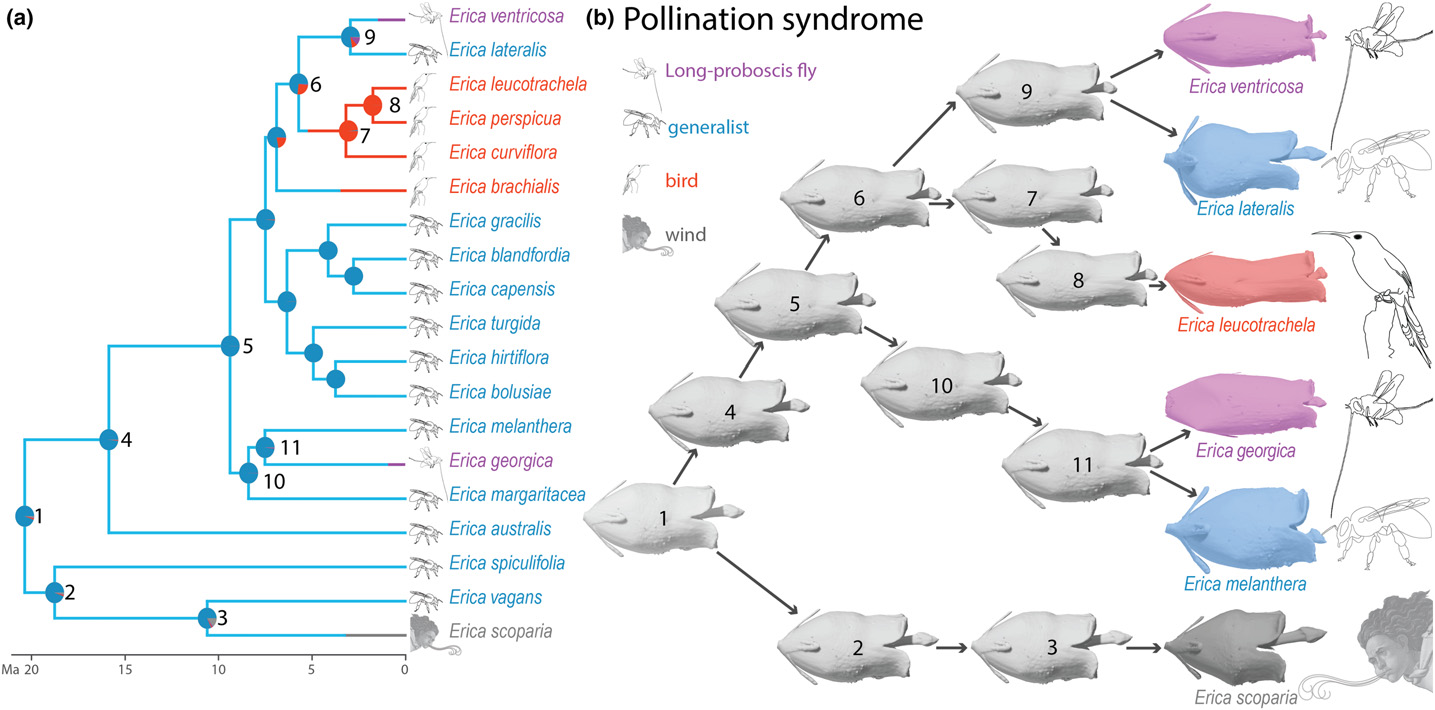
Ancestral state reconstruction for pollination syndromes and floral shape in *Erica*. (a) Stochastic character mapping of the four pollination syndromes optimised on a chronogram inferred from Bayesian dating. Pie charts at internal nodes indicate the proportion of stochastic mapping from 1000 runs using the Equal Rates (ER) model. (b) Ancestral shape reconstruction and reconstructed evolutionary trajectories for six selected species of *Erica*, including species from all four studied pollination syndromes and two convergent evolution of flowers with long‐proboscid fly syndrome.

Ancestral state reconstruction for pollination syndromes (Fig. 5a) suggests that the generalist pollination syndrome is the possible most recent common ancestral (MRCA) state in *Erica*. Within our sampled species the bird pollination syndrome, as well as the LPF syndrome evolved twice independently.

### Evolution of whole‐flower shape

Under the penalized likelihood approach, the best‐fitting model to the evolution of the highly‐dimensional whole floral shape in *Erica* was the Ornstein–Uhlenbeck model (OU; lowest GIC; Table S9), which assumes evolution towards an optimal floral shape mean as would be expected under selection. The MRCA floral shape of *Erica* most likely displays short and urceolate flowers (Fig. 5b, node 1). The reconstructed evolutionary trajectory (under the best‐fitted model of OU) displays likely late differentiations in flower shape, with most differentiation possibly occurring at the most recent internal nodes of the tree (Fig. 5b, nodes 3, 7–9). In the most recent internal nodes of ancestors of the two species with LPF syndrome (Fig. 5b, nodes 9, 11), the reconstructed ancestral shape displays differentiation as compared to more internal nodes (e.g. Fig. 5b, nodes 5, 6, 10), but this differentiation is weak compared to that of the terminal nodes (Fig. 5b, flowers of *E. ventricosa* and *E. georgica*).

### Evolution of individual‐flower traits

The results of the fitting of five models (BM_1_, BM_S_, OU_1_, OU_M_ and OU_MV_) on quantitative floral trait evolution (shape PC1–5, size and integration) under the four pollination‐syndrome regimes are summarized in Table 3. The Hessian matrix of one model (i.e. OU_MV_) displayed a negative eigenvalue for PC3, PC4, integration and centroid size, which means that this model was too complex for the information contained in these data and it was excluded from the analyses. Different evolutionary models yielded variable AICc distributions and AICc weights (see Table 3). The evolution of floral shape along PC1 and centroid size of flowers best‐fitted an OU_M_ model (see Table 3), which suggests selection around four different optimal values, one per pollination syndrome (see θ values in Table S10). This suggests that PC1 and centroid size have different evolutionary means for each of the four pollination syndrome regimes and that there is an evolutionary force that maintains PC1 and size closer to this evolutionary mean than would be expected under a drift‐like (BM) model. The evolution of floral shape along PC2 and PC5, and floral integration were found to best‐fit an OU_1_ model (see Table 3). This result suggests that there is no difference between the four pollination syndromes, and that PC2, PC5 and integration each evolve towards one single optimal value across all *Erica* species (see θ values in Table S10), indicating no evidence for different constraints by the four pollination regimes. The best‐fittin model for the evolution of floral shape along PC3 and PC4 was a BM_1_ model (see Table 3); this model where there is no difference between the pollination syndromes, and these floral variables evolve according to a random walk process.

**Table 3:** Models of quantitative phenotypic trait evolution (PC1–5 of floral shape, size and integration) under the pollination syndrome regime, and their biological interpretation, model fit of plausible models for the seven floral variables, indicating AICc (corrected AIC score) and AICc weight (best‐supported model in bold).

Variables	Model	AICc	AICc weight	Interpretation of the best model for shape, integration and size variable evolution
PC1	BM1	−6.65	0.015	Evolution of shape along PC1 is constrained; different optima depend on pollination syndromes, which would imply that optimal shape along PC1 has evolved separately for different pollination syndromes
	BMS	0.46	4.22E‐04	
	OU1	−5.5	0.008	
	**OUM**	**−14.56**	**0.772**	
	OUMV	−11.9	0.204	
PC2	BM	−11.31	0.232	Evolution of shape along PC2 is directed toward an optimum without being affected by the pollination syndromes
	BMS	−4.76	0.009	
	**OU1**	**−13.42**	**0.667**	
	OUM	−9.44	0.091	
	OUMV	−1.27	0.002	
PC3	**BM**	**−34.98**	**0.758**	Evolution of shape along PC3 is random and not affected by the different pollination syndromes
	BMS	−26.25	0.010	
	OU1	−32.61	0.231	
	OUM	−22.69	0.002	
PC4	**BM**	**−38.35**	**0.630**	Evolution of shape along PC4 is random and not affected by the different pollination syndromes
	BMS	−30.17	0.011	
	OU1	−37.03	0.326	
	OUM	−32.5	0.034	
PC5	BM	−37.62	0.437	Evolution of shape along PC5 is directed toward an optimum without being affected by the pollination syndromes
	BMS	−30.49	0.012	
	**OU1**	**−38.01**	**0.533**	
	OUM	−31.22	0.018	
	OUMV	−18.84	3.66E‐05	
Integration	BM	−47.52	0.364	Evolution of shape integration is directed toward an optimum without being affected by the pollination syndromes
	BMS	−38.67	0.004	
	**OU1**	**−48.61**	**0.6264**	
	OUM	−39.22	0.006	
Centroid size	BM1	160.28	9.42E‐06	Evolution of size is constrained; different optima depend on pollination syndromes, which would imply that optimal size has evolved separately for different pollination syndromes
	BMS	144.98	0.020	
	OU1	161.09	6.29E‐06	
	**OUM**	**137.17**	**0.98**	

## Discussion

### Generalist syndrome: trade‐off selection and developmental modularity

In flowers with the generalist syndrome, our observation of strong support for the *developmental modularity* hypothesis supports the ‘trade‐off’ hypothesis of evolution of generalist flowers (which implies the absence of functional modules), and invalidates both the ‘trait specialisation’ and ‘common shape’ hypotheses (which both imply the evolution of functional modules). Our findings contrast with most published studies that recover support for the functional hypothesis in flowers with generalist syndrome (see, e.g. Conner & Via, 1993; Conner & Sterling, 1995; Conner, 1997, 2002; Sánchez‐Lafuente 2002; Armbruster *et al.*, 2004; Pérez‐Barrales *et al.*, 2007; Pérez *et al.*, 2007; Rosas‐Guerrero *et al.*, 2011; Fornoni *et al.*, 2015; Armbruster & Wege, 2018). This discrepancy is most likely a consequence of methodological differences: most studies include seven or fewer metrics (mostly organs’ lengths), which implies that organs categories are represented by one or two metrics. Within such a system of metrics, some organ categories will be represented by only one metric; there is therefore little to no possibility to contrast correlations within and among organs. With such metrics, support for the developmental hypothesis will only be identified if correlation among organs is low. Geometric morphometrics (GM) allows us to study flower shape and its evolution at a higher level of detail than with distance‐based metrics: with GM based‐metrics we can contrast multiple correlations among and within organs and identify developmental modularity, even in the case of high correlations among modules. It is therefore possible that the prevalence of functional modules has been overestimated.

### Specialized syndromes

Our findings for the generalist syndrome contrast with our findings for flowers with specialized syndromes (bird and LPF syndromes) which display support for (different) *functional* hypotheses. Similar patterns of modularity to that supported in flowers with bird syndrome (attraction–receipt–deposition) have been reported widely (see, e.g. Conner & Sterling, 1995; Cresswell, 1998; Armbruster *et al.*, 2004; Anderson & Busch, 2006; Pérez‐Barrales *et al.*, 2007; Bissell & Diggle, 2008, 2010; Nattero *et al.*, 2010; Rosas‐Guerrero *et al.*, 2011; Wanderley *et al.*, 2016; Heywood *et al.*, 2017).

### Bird syndrome: pollen deposition/receipt and development

Both the functional hypothesis 1 (pollen deposition/receipt) and the developmental hypothesis are well supported in bird‐pollinated flowers; this supports the hypothesis that developmental modularity is a default modularity, a form of null hypothesis (Herrera, 2001). The high support for both hypotheses also likely indicates that most independence is between the anthers and the stigma. Moreover, the high support for the developmental hypothesis makes it likely that a distance‐based approach would have supported a developmental modularity. Identifying the correct pollen receipt and deposition modules would have required us to carry out measurements of the distance from the corolla base to the adaxial median meeting point of the corolla lobes and the stigma, and contrast these measures with the distance from the corolla base to the other three meeting point of the corolla lobes, and include detailed measurements of the corolla mouth, and stamen lengths to contrast with other measurements of the corolla and sepals. Identifying these modules would have been very difficult without the use of GM.

### LPF syndrome: restriction and spandrel

In the flowers with LFP syndrome, the set of landmarks of the ‘corolla aperture’ does not include any reproductive organs; the function of this set is thus most likely not directly pollen deposition or receipt. In the Cape, flowers with LPF syndrome typically have very narrow floral tubes (Goldblatt & Manning, 2000). *Erica* flowers with this syndrome, however, do not always have narrow tubes, but do have a narrow corolla apertures (see Fig. 4c) (Rebelo *et al.*, 1985). This corolla aperture likely plays a role in restricting access to the floral rewards to illegitimate visitors. This interpretation is supported by the allometric shape deformation (how shape changes with size) in LPF syndrome flowers: in shape, in larger flowers the corolla aperture is, relative to the rest of the flower, narrower, but in size, the corolla aperture stays about the same size in smaller and in larger flowers (Fig. S3b). Because of its putative function, we propose to refer to the set of landmarks on the corolla aperture as a ‘restriction module’. Similar structures were found to preclude visits from bats in bird‐pollinated *Burmeistera* (Campanulaceae) and to vary much less than the rest of the flower (Muchhala, 2006), suggesting that they constitute an independent module. Moreover, this restriction module also contains the petal tips (Fig. 4c), that do not actively contribute to limiting access to the floral reward. The small size of the corolla lobes relative to the rest of the corolla also precludes a major role in pollinator attraction. Their presence in the restriction module is therefore most likely nonadaptive and only due to their developmental proximity to the corolla aperture. Their presence within the restriction module is therefore most likely an evolutionary spandrel *sensu* Gould and Lewontin (Gould & Lewontin, 1979): a fundamental but nonadaptive architectural constraint of *Erica* flowers. If it were feasible, a denser sampling of landmarks across the flowers would probably uncover more of such structures grouping in shape modules owing to their developmental proximity and not their function.

### Wind syndrome: developmental modularity and allometry

In flowers with wind syndrome, support for the developmental hypothesis suggests that the shape of the different organ classes is independent from each other. This could be due to the fact that (1) wind‐pollinated flowers probably do not require modules across organ classes (for pollen receipt), and (2) that our data are dominated by developmental shape changes. Modelling studies in grasses that have shown that pollen deposition overwhelmingly relies on direct impact on the stigma and not on air flows generated by the rest of the flower (Cresswell *et al.*, 2010); this suggests that there is no selection pressure for the rest of the flower to form pollen receipt modules (as in *functional* hypothesis 1). The strong but weakly significant allometry reflects typical differences in flower shape related to differences in anthesis stage: larger (older) flowers have more open petals and more exerted stamens than smaller (younger) flowers (Fig. S3c); these changes also would cause organs classes to each display shape variation along their own developmental axis and be independent from each other. This notwithstanding, any interpretation is tentative given our limited sampling of this syndrome.

### Evolution of flower shape

The radiation of *Erica* in the Cape is the greatest floral radiation known to have occurred there and one of the greatest in recent plant evolutionary history (Pirie *et al.*, 2016). Analyses confirmed the ‘hotbed’ hypothesis in the genus (i.e. that the radiation of *Erica* was due to increased speciation rates) and showed an overall recent slowing down of speciation rates (although they do remain high in the former South Western clade; Pirie *et al.*, 2016). Shifts in multiple local‐scale ecological gradients, and repeated shifts in plants' preferences for pollinators appear to have taken place (Linder *et al.*, 2010; Pirie *et al.*, 2016). Such a radiation fits Simpson's adaptive zone model in which similar niches become ecologically available to a lineage, free from competitors (Simpson, 1944): when a lineage first enters these zones, phenotypical evolution should at first be fast, but as ecological niches are filled, the rate of phenotypical evolution should then slow down (Simpson, 1944; Schluter, 2000; Losos & Miles, 2002; Harmon *et al.*, 2010). In such a radiation, one would expect to recover an Early Burst (EB) mode of phenotypical evolution (Harmon *et al.*, 2010). However, our analysis of the highly‐dimensional morphometric dataset of flower shape best‐fitted an Ornstein–Uhlenbeck (OU) model of evolution (Table S9), a model considered to better represent the importance of selection. This is further supported by our ancestral floral shape reconstruction (Fig. 5b), which indicates a pattern of greater phenotypical variation at the most recent internal nodes of the tree (Fig. 5a,b, nodes 3, 8, 9, 11), a pattern consistent with pollinator‐driven selection (OU model; Harmon *et al.*, 2010). Our finding of strong evolutionary changes over short timescales concurs with previous findings from diverse data sources (Gingerich, 1983; Lynch, 1990; Hendry & Kinnison, 1999; Roopnarine, 2003; Estes & Arnold, 2007; Harmon *et al.*, 2010). This is furthermore strongly supported by our analyses of the evolutionary model of Principal Component (PC)1 and centroid size under different regimes (i.e. pollination syndrome) which recovered as best fit an OU_M_ model of evolution (selection towards different optima; Tables 3, S10), strongly indicating that pollinators have indeed driven the evolution of floral shape (see below), therefore supporting a strong role for pollinator‐driven speciation in *Erica* (Pirie *et al.*, 2011). PC1 corresponds to a shape change from open bell‐shaped flowers to more elongated, tubular flowers, generating, for the same size, longer tubes and strongly affecting the landmarks on the narrowest and broadest parts of the corolla (see Fig. 3). These landmarks, together with tube length, were shown by our random forest analyses to be especially important in predicting pollination syndromes (see Table S6). PC1 therefore involves a shape change that is especially relevant for the generation of the different floral shapes of the different pollination syndromes. Variation in PC1 was thus most likely co‐opted by evolution to generate the different syndrome morphologies, and ended up encapsulating almost 40% of shape variance (Table S10). Likewise, centroid size is strongly correlated with tube length (*R* ^2^ = 0.96; *P* = 2.2E–16; *n* = 209), the variable we demonstrate to play the strongest role in predicting the different syndromes (Fig. S1a; Table S6). Other PCs probably do not generate variation for which divergent selection on syndromes was present (or strong enough to be identified with our limited sampling), and therefore follow either a single optimum (OU_1_) or a random model (Brownian Motion (BM)_1_) of evolution (Tables 3, S10).

### Evolution of integration

Our results demonstrate that integration follows an OU_1_ model of evolution (which means that there is selection towards a single optimum; Tables 3, S10). This does not support increased floral integration in specialist compared to generalist flowers. Our results also contrast with the results of Gomez *et al.* (2014), who recovered a BM model of evolution for floral integration (Gomez *et al.*, 2014). However, Gomez *et al.* (2014) included only landmarks placed on the petals (in 2D), whereas our study includes reproductive organs (in 3D). Because functional modularity (including reproductive parts) has been shown to be stronger than attraction modularity (including the petals only) (Rosas‐Guerrero *et al.*, 2011), our study likely includes a signal that is not present in that of Gomez *et al.* (2014). Evolution of whole‐flower integration towards a single optimum suggests that evolution of increased integration in functional part of the flowers may come at the cost of lower integration with other parts of the flowers, leading to evolution towards a single optimal value in generalized and specialized systems. Our findings thus do not support changes in integration as a whole, but strongly support changes in its structure, an observation congruent with (Ordano *et al.*, 2008).

### Outlook

Our results illustrate for the first time the potential of 3D landmark datasets (that include the reproductive organs of flowers) together with geometric morphometrics to uncover the modularity of the highly dimensional shape of flowers as a function of pollinator syndrome, and together with a novel penalized likelihood framework (Clavel *et al.*, 2018) also for the first time to test the fits of evolutionary models to the macro‐evolution of high‐dimensional flower shape and reconstruct its trajectory. Together, these new approaches open new perspectives to the study of flower shape modularity integration and evolution.

## Author contributions

YMS, MvB and SM designed the project; YMS and MvB collected the material; JS provided access to the equipment and technical staff; DR and AB collected the data and ran preliminary analyses; MC carried out the random forest analyses; SM and MS carried out the phylogenetic analyses; SM carried out the trait evolution analyses; YMS carried out the geometric morphometric analyses; SM designed the manuscript; and YMS and SM wrote the manuscript. DR and AB contributed equally to this work.

## Supporting information

Please note: Wiley Blackwell are not responsible for the content or functionality of any Supporting Information supplied by the authors. Any queries (other than missing material) should be directed to the *New Phytologist* Central Office.

**Fig. S1** Machine learning.
**Fig. S2** Hypotheses test: RV distributions.
**Fig. S3** Allometry in *Erica* flowers.

**Methods S1** Details of the methodologies used to for: X‐ray tomography, 3D‐landmarking, geometric morphometrics, pollination syndrome prediction, modularity analyses (exploratory and confirmatory approaches), phylogenetic inference, ancestral character states reconstruction and models of trait evolution.
**Notes S1** Literature analysis.
**Notes S2** Pollination observation *Erica gracillis* in cultivation, allometric regressions, and correlation between the corolla tube length and centroid size.

**Table S1** Species, sample numbers (*n*) and scanning conditions of *Erica* flowers.
**Table S2** Landmarks used to digitize the shape of *Erica* flowers, and modules to which they belong in the modularity hypotheses tested.
**Table S3** Species‐level average values for size and integration.
**Table S4** Genbank accession numbers for nrDNA ITS and cpDNA trnL‐F‐ndhJ and trnT‐L sequence data.
**Table S5** Discrete character‐mapping models for pollination syndromes.
**Table S6** Main variables mean accuracy decrease of RF syndrome prediction.
**Table S7** Corolla tube length per flower.
**Table S8** Classification of 114 individual flowers of diverse *Erica* species into the pollination syndromes.
**Table S9** Support values for evolutionary models of floral shape evolution.
**Table S10** Summary of the preferred models of evolution for seven phenotypic trait variables (PC1‐5 of floral shape, centroid size and integration.

## Data Availability

All of the scan data are deposited on PHAIDRA, the open data repository of the University of Vienna (https://phaidra.univie.ac.at/o:10465900.
