# Supplementary material for: Modularity and evolution of flower shape: the role of function, development, and spandrels in Erica
Source: New Phytol. 2020 Jan 8;226(1):267–80. doi: 10.1111/nph.16337 (PMC7065081; doi:10.1111/nph.16337)

New Phytologist Supporting Information

Manuscript title: Modularity and evolution of flower shape: the role of function, development, and spandrels in *Erica*

Authors: Dieter Reich, Andreas Berger, Maria von Balthazar, Marion Chartier, Mahboubeh Sherafati, Jürg Schönenberger, Sara Manafzadeh, Yannick M. Staedler

Acceptance date: 10 November 2019

The following supporting figures are available for this article:

Figure S1 Machine learning. (a) landmark coordinates and tube length sorted by mean accuracy decrease in predicting pollination syndrome via Random Forest (the tube length is the best variable to predict pollination syndrome). (b) tube length (in mm) in studied species.

Figure S2 Hypotheses test: RV distributions. X-axis RV coefficient, y-axis frequency of values. Red arrow indicates the value of the RV coefficient of the modularity hypothesis tested. Left, hypotheses tested, right results of test.

Figure S3 Allometry in *Erica* flowers. (a) allometric plot. x-axis, log centroid size, y-axis shape axis. (b) allometric deformation in flowers with long-proboscid syndrome. (c) allometric deformation in flowers with wind syndrome.

Figure S1 Machine learning. (a) landmark coordinates and tube length sorted by mean accuracy decrease in predicting pollination syndrome via Random Forest (the tube length is the best variable to predict pollination syndrome). (b) tube length (in mm) in studied species.

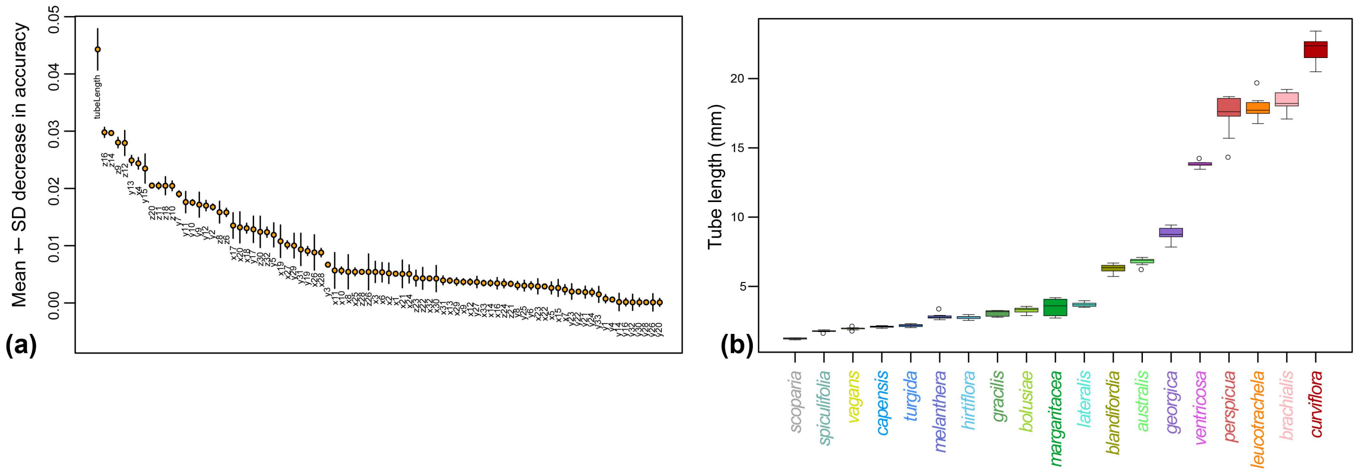

Figure S2 Hypotheses test: RV distributions. X-axis RV coefficient, y-axis frequency of values. Red arrow indicates the value of the RV coefficient of the modularity hypothesis tested. Left, hypotheses tested, right results of test. a-d tests for species with generalist syndrome. a, test of *attraction-reproduction* hypothesis. b, test for *functional* hypothesis 1. c, test for *functional* hypothesis 2. d, test for *developmental* hypothesis. e-h tests for species with bird syndrome. e, test of *attraction-reproduction* hypothesis. f, test for *functional* hypothesis 1. g, test for *functional* hypothesis 2. h, test for *developmental* hypothesis. i-l tests for species with long-proboscid fly syndrome. i, test of *attraction-reproduction* hypothesis. j, test for *functional* hypothesis 1. k, test for *functional* hypothesis 2. l, test for *developmental* hypothesis. m-p tests for species with wind syndrome. m, test of *attraction-reproduction* hypothesis. n, test for *functional* hypothesis 1. o, test for *functional* hypothesis 2. p, test for *developmental* hypothesis. Pollinator drawings, originals. Generalists represented by drawing of bee. Character representing the wind: Zephyr from "The birth of Venus" by Sandro Boticelli (ca. 1480).

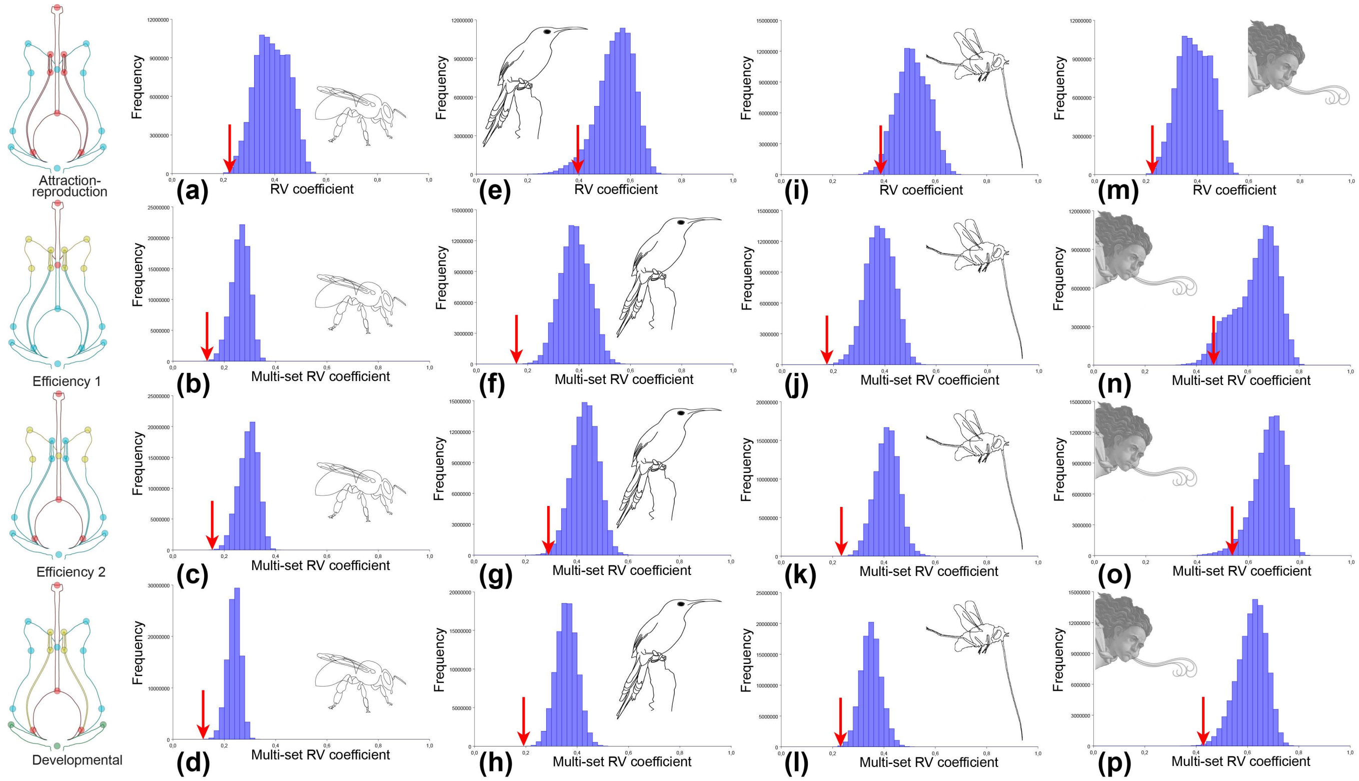

Figure S3 Allometry in *Erica* flowers. (a) allometric plot. x-axis, log centroid size, y-axis shape axis. All 209 individual flowers from all 19 species studied are plotted. Blue to green dots generalist syndrome, orange to red squares bird syndrome, pink and purple triangles long-proboscid fly syndrome, gray crosses wind syndrome. (b) allometric deformation in flowers with long-proboscid syndrome for a change in log centroid size of 0.2. Pink, schematic drawing of smaller flowers, blue schematic drawing of larger flowers. (c) allometric deformation in flowers with wind syndrome for a change in log centroid size of 0.2. Pink, schematic drawing of smaller flowers, blue schematic drawing of larger flowers.

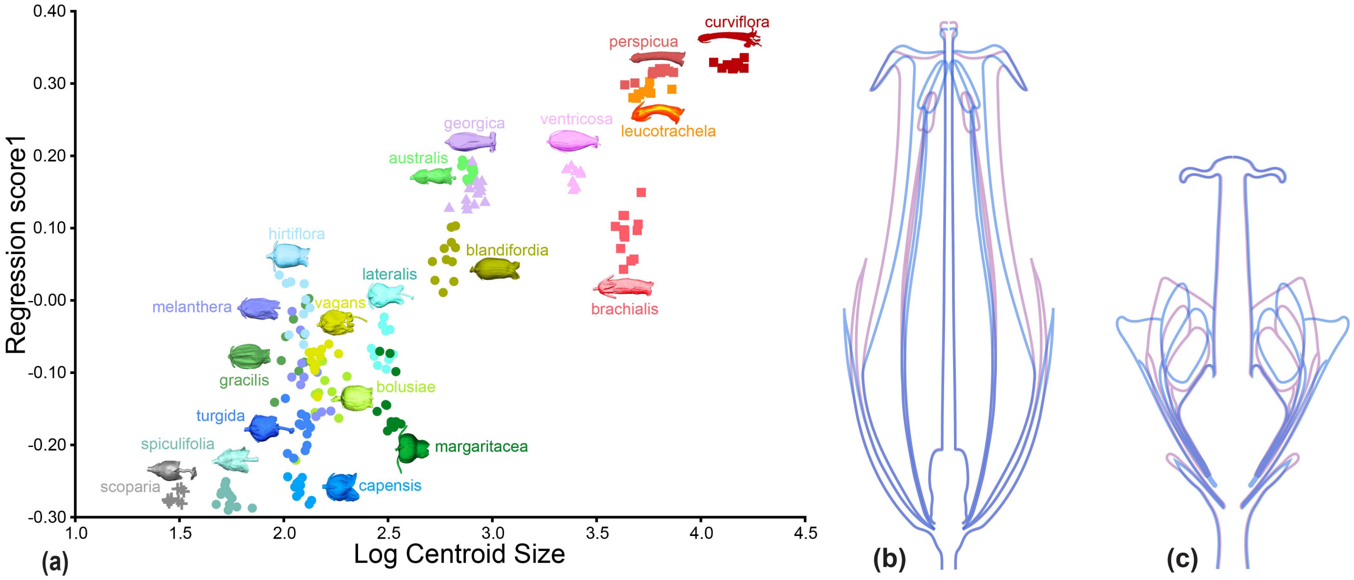

Supplement: Supplementary file 1 — Fig. S1 Machine learning. Fig. S2 Hypotheses test: RV distributions. Fig. S3 Allometry in Erica flowers. [file NPH-226-267-s001.pdf]
