# Supplementary material for: Modularity and evolution of flower shape: the role of function, development, and spandrels in Erica
Source: New Phytol. 2020 Jan 8;226(1):267–80. doi: 10.1111/nph.16337 (PMC7065081; doi:10.1111/nph.16337)
Supplement: Supplementary file 3 — Table S1 Species, sample numbers (n) and scanning conditions of Erica flowers. Table S2 Landmarks used to digitize the shape of Erica flowers, and modules to which they belong in the modularity hypotheses tested. Table S3 Species‐level average values for size and integration. Table S4 Genbank accession numbers for nrDNA ITS and cpDNA trnL‐F‐ndhJ and trnT‐L sequence data. Table S5 Discrete character‐mapping models for pollination syndromes. Table S6 Main variables mean accuracy decrease of RF syndrome prediction. Table S7 Corolla tube length per flower. Table S8 Classification of 114 individual flowers of diverse Erica species into the pollination syndromes. Table S9 Support values for evolutionary models of floral shape evolution. Table S10 Summary of the preferred models of evolution for seven phenotypic trait variables (PC1‐5 of floral shape, centroid size and integration. [file NPH-226-267-s003.pdf]

New Phytologist Supporting Information

Manuscript title: Modularity and evolution of flower shape: the role of function, development, and spandrels in *Erica*

Authors: Dieter Reich, Andreas Berger, Maria von Balthazar, Marion Chartier, Mahboubeh Sherafati, Jürg Schönenberger, Sara Manafzadeh, Yannick M. Staedler

Acceptance date: 10 November 2019

The following supporting tables are available for this article:

Table S1. Species, sample numbers (n) and scanning conditions of *Erica* flowers.
Table S2. Landmarks used to digitise the shape of *Erica* flowers, and modules to which they belong in the modularity hypotheses tested.
Table S3. Species-level average values for size and integration.
Table S4. Genbank accession numbers for nrDNA ITS and cpDNA trnL-F-ndhJ and trnT-L sequence data.
Table S5. Discrete character mapping models for pollination syndromes.
Table S6. Main variables mean accuracy decrease of random forest syndrome prediction.
Table S7. Corolla tube length per flower.
Table S8. Classification of 114 individual flowers of diverse *Erica* species into the pollination syndromes,
Table S9. Support values evolutionary models of floral shape evolution.
Table S10. Summary of the preferred models of evolution for seven phenotypic trait variables (PC1-5 of floral shape, centroid size, and integration

Table S1. Species, sample numbers (n) and scanning conditions of *Erica* flowers. LFOV, large field of view objective. Voltage and current parameters refer to X-Ray source acceleration voltage and current, respectively.

sample	sample	species	n	Voltage [kV]	Current [μA]	Exposure time [s]	Pictures /sample	Pixel size [μm]	Objective	Binning
batch1	A + B	*E. brachialis*	8	40	200	6	728	15.5	LFOV	1
batch2	A	*E. curviflora*	4	40	200	5	728	17.7	LFOV	1
batch2	B	*E. curviflora*	3	40	200	5	728	18.4	LFOV	1
batch3	C	*E. perspicua*	4	40	200	5	728	13.1	LFOV	1
batch3	B	*E. curvirostris*	6	40	200	5	728	11.0	LFOV	1
batch3	A	*E. leucotrachela*	4	40	200	5	728	14.9	LFOV	1
batch4	C	*E. curvirostris*	6	40	200	5	728	17.4	LFOV	1
batch4	B	*E. curviflora*	4	40	200	5	728	19.0	LFOV	1
batch4	A	*E. brachialis*	4	40	200	6	728	17.4	LFOV	1
batch5	D+ E	*E. melanthera*	13	40	200	5	728	11.3	LFOV	1
batch5	C	*E. blandfordia*	5	40	200	5	728	11.3	LFOV	1
batch5	B	*E. hirtiflora*	7	40	200	5	728	9.1	LFOV	1
batch5	A	*E. lateralis*	6	40	200	5	728	11.3	LFOV	1
batch6	E	*E. turgida*	8	40	200	5	728	10.3	LFOV	1
batch6	D	*E. lateralis*	5	40	200	5	728	10.3	LFOV	1
batch6	C	*E. turgida*	7	40	200	5	728	9.1	LFOV	1
batch6	B	*E. blandfordia*	5	40	200	5	728	10.5	LFOV	1
batch6	A	*E. blandfordia*	4	40	200	5	728	11.5	LFOV	1
batch7	A + B	*E. leucotrachela*	8	40	200	5	728	17.8	LFOV	1
batch8	C	*E. perspicua*	4	40	200	6	728	12.7	LFOV	1
batch8	A + B	*E. ventricosa*	8	40	200	5	728	12.7	LFOV	1
batch9	D+E	*E. gracilis*	12	40	200	6	728	10.5	LFOV	1
batch9	C	*E. hirtiflora*	7	40	200	6	728	10.5	LFOV	1
batch9	B	*E. capensis*	6	40	200	6	728	10.5	LFOV	1
batch9	A	*E. capensis*	6	40	200	6	728	9.9	LFOV	1
batch11	C	*E. ventricosa*	4	40	200	6	728	12.7	LFOV	1
batch11	B	*E. perspicua*	4	40	200	6	728	13.3	LFOV	1
batch12	B	*E. gracilis*	6	40	200	6	728	9.5	1x	1
batch12	A	*E. gracilis*	6	40	200	12	728	10.6	1x	1
batch13	C	*E. spiculifolia*	8	40	200	6	728	7.0	LFOV	1
batch13	B	*E. australis*	4	40	200	4	728	10.9	LFOV	1
batch13	A	*E. australis*	8	40	200	3	728	13.1	LFOV	1
batch14	E	*E. spiculifolia*	8	40	200	15	728	9.7	LFOV	1
batch14	D	*E. scoparia*	5	40	200	15	728	9.7	LFOV	1
batch14	A+B+C	*E. georgica*	15	40	200	5	728	9.7	LFOV	1
batch15	E	*E. scoparia*	8	40	200	5	728	9.2	LFOV	1
batch15	D	*E. vagans*	7	40	200	5	728	8.8	LFOV	1
batch15	C	*E. vagans*	7	40	200	5	728	8.7	LFOV	1
batch15	B	*E. melanthera*	6	40	200	5	728	8.4	LFOV	1
batch15	A	*E. melanthera*	7	40	200	5	728	8.8	LFOV	1

Table S2. Landmarks used to digitise the shape of *Erica* flowers (number, flower part, and position in frontal view), and modules to which they belong in the modularity hypotheses tested.

landmark number	flower part	position in frontal view	attraction/ reproduction	developmental	efficiency 1	efficiency 2
1	base of flower		reproduction	carpels	remainder	carpels
2	nectary	upper	reproduction	carpels	remainder	carpels
3	nectary	lower	reproduction	carpels	remainder	carpels
4	ovary/style transition		reproduction	carpels	remainder	carpels
5	sepal tip	upper	attraction	sepals	remainder	remainder
6	sepal tip	left	attraction	sepals	remainder	remainder
7	sepal tip	lower	attraction	sepals	remainder	remainder
8	sepal tip	right	attraction	sepals	remainder	remainder
9	corolla - widest position	upper/left	attraction	petals	remainder	remainder
10	corolla - widest position	lower/left	attraction	petals	remainder	remainder
11	corolla - widest position	lower/right	attraction	petals	remainder	remainder
12	corolla - widest position	upper/right	attraction	petals	remainder	remainder
13	corolla - narrowest position	upper	attraction	petals	remainder	aperture
14	corolla - narrowest position	left	attraction	petals	remainder	aperture
15	corolla - narrowest position	lower	attraction	petals	remainder	aperture
16	corolla - narrowest position	right	attraction	petals	remainder	aperture
17	meeting of petal lobes	upper	attraction	petals	deposition	aperture
18	meeting of petal lobes	left	attraction	petals	deposition	aperture
19	meeting of petal lobes	lower	attraction	petals	receipt	aperture
20	meeting of petal lobes	right	attraction	petals	deposition	aperture
21	petal tip	upper/left	attraction	stamens	deposition	aperture
22	petal tip	lower/left	attraction	stamens	deposition	aperture
23	petal tip	lower/right	attraction	stamens	deposition	aperture
24	petal tip	upper/right	attraction	stamens	deposition	aperture
25	anther tip	upper	reproduction	stamens	deposition	remainder
26	anther tip	left	reproduction	stamens	deposition	remainder
27	anther tip	lower	reproduction	stamens	deposition	remainder
28	anther tip	right	reproduction	stamens	deposition	remainder
29	anther base	upper	reproduction	stamens	deposition	remainder
30	anther base	left	reproduction	stamens	deposition	remainder
31	anther base	lower	reproduction	stamens	deposition	remainder
32	anther base	right	reproduction	stamens	deposition	remainder
33	tip of stigma		reproduction	carpels	receipt	carpels

Table S3. Species-level average values for size (centroid size) and integration (eigenvalue variance scaled by total variance and number of variables).

Species	Centroid size	Integration
Erica australis L.	18,0	0,20
Erica blandfordia Andrews	16,2	0,17
Erica bolusiae T. M. Salter	8,9	0,24
Erica brachialis Salisb.	38,4	0,22
Erica capensis T.M. Salter	8,0	0,17
Erica curviflora L.	63,6	0,19
Erica georgica L. Guthrie & Bolus	18,3	0,14
Erica gracilis J.C. Wendl.	7,9	0,18
Erica hirtiflora Curtis	7,9	0,29
Erica lateralis Willd.	11,9	0,16
Erica leucotrachela H.A. Baker	42,0	0,21
Erica margaritacea Aiton	12,3	0,32
Erica melanthera L.	8,3	0,33
Erica perspicua J.C. Wendl.	44,0	0,16
Erica scoparia L.	4,4	0,24
Erica spiculifolia Salisb.	5,7	0,26
Erica turgida Salisb.	8,0	0,14
Erica vagans L.	8,7	0,27
Erica ventricosa Thunb.	29,8	0,23

Table S4. Genbank accession numbers for nrDNA ITS and cpDNA trnL-F-ndhJ and trnT-L sequence data. The dash symbol (–) shows that the sequence is not available.

**Species**	**Source or Specimen**	**ITS**	***trn*LF-ndhJ**	***trnT-L***
Calluna vulgaris	Pirie et al., 2016	HQ858882	KP737378	_
Daboecia cantabrica	Pirie et al., 2016	HQ859000	KP737380	KP737653
Erica australis	Pirie et al., 2016	HQ858926	HQ858927	HQ858928
Erica blandfordii	Pirie et al., 2016	HQ858940	KU832634	KU831854
Erica bolusiae	Pirie et al., 2016	KU832354	KU832640	KU831859
Erica brachialis	Pirie et al., 2016	HQ858944	KU832642	KU831861
Erica capensis	Pirie et al., 2016	KU832452	KU832846	KU832055
Erica curviflora	Pirie et al., 2016	KU832381	KU832698	KU831913
Erica georgica	Pirie et al., 2016	KU832410	KU832764	KU831977
Erica gracilis	Pirie et al., 2016	HQ859074	KU832788	KU832000
Erica hirtiflora	Pirie et al., 2016	HQ859084	KU832808	KU832019
Erica lateralis	Pirie et al., 2016	KU832456	KU832851	KU832060
Erica leucotrachela	Pirie et al., 2016	KU832461	KU832865	_
Erica margaritacea	Pirie et al., 2016	KU832468	KU832882	KU832088
Erica melanthera	Pirie et al., 2016	HQ859145	KU832889	KU832094
Erica perspicua	Pirie et al., 2016	HQ859192	KU832949	KU832153
Erica scoparia	Mugrabi de Kuppler et al., 2015	KP737593	KP737459	KP737674
Erica spiculifolia	Mugrabi de Kuppler et al., 2015	KP737610	KP737475	KP737678
Erica turgida	Pirie et al., 2016	HQ859312	KU833085	KU832288
Erica vagans	Pirie et al., 2016	HQ859319	KP737490	KP737682
Erica ventricosa	Pirie et al., 2016	KU832562	KU833104	KU832307

Table S5. Discrete character mapping models for pollination syndromes. Model testing (equal-rates model – ER, symmetrical rates model – SYM and all-rates-different model – ARD) for ancestral state reconstruction under the ML approach. The ER model is preferred (lowest AICc and AICc Weight values). AICc = corrected AIC score.

	AICc	AICc Weight
**ER**	**39.1**	**0.99955**
SYM	54.6	0.00045
ARD	108	0

Table S6. Main variables mean accuracy decrease of random forest syndrome prediction (averaged on 500 RF made of 1001 trees each).

Variable	Mean (± SD) decrease in accuracy
tube length	0.0442 ± 0.0036
z16 - corolla narrowest position	0.0296 ± 0.0031
z14 - corolla narrowest position	0.0297 ± 0.0031
z9 - corolla widest position	0.0279 ± 0.0027
z12 - corolla widest position	0.0281 ± 0.0028
y13 - corolla narrowest position	0.0248 ± 0.0031
x4 - ovary/style transition	0.0248 ± 0.0025
y15 - corolla narrowest position	0.0234 ± 0.0030
z20 - meeting of petal lobes	0.0204 ± 0.0001
z11 - corolla widest position	0.0204 ± 0.0028
z18 - meeting of petal lobes	0.0206 ± 0.0024
z10 - corolla widest position	0.0206 ± 0.0023
y7 - sepal tip	0.0190 ± 0.0022
y11 - corolla widest position	0.0176 ± 0.0021
y10 - corolla widest position	0.0175 ± 0.0023
y9 - corolla widest position	0.0172 ± 0.0022

Table S7. Corolla tube length per flower calculated as the distance between the base of the flower and the average position of the meeting of the four corolla lobes.

flower number	*Erica* species	tube length
1	*australis*	6.27
2	*australis*	6.92
3	*australis*	6.58
4	*australis*	6.99
5	*australis*	7.03
6	*australis*	7.10
7	*australis*	6.82
8	*australis*	6.57
9	*australis*	6.88
10	*australis*	6.85
11	*australis*	6.87
12	*capensis*	2.10
13	*capensis*	2.09
14	*capensis*	2.13
15	*capensis*	2.07
16	*capensis*	2.05
17	*capensis*	2.17
18	*capensis*	2.15
19	*capensis*	2.06
20	*capensis*	1.97
21	*capensis*	2.13
22	*blandifordia*	6.23
23	*blandifordia*	6.62
24	*blandifordia*	6.03
25	*blandifordia*	6.38
26	*blandifordia*	6.06
27	*blandifordia*	6.69
28	*blandifordia*	6.63
29	*blandifordia*	6.17
30	*blandifordia*	6.35
31	*blandifordia*	6.44
32	*blandifordia*	5.72
33	*brachialis*	18.21
34	*brachialis*	18.97
35	*brachialis*	18.07
36	*brachialis*	18.91

37	*brachialis*	18.19
38	*brachialis*	17.97
39	*brachialis*	19.21
40	*brachialis*	18.05
41	*brachialis*	19.13
42	*brachialis*	19.16
43	*brachialis*	18.02
44	*brachialis*	17.08
45	*brachialis*	17.97
46	*brachialis*	18.36
47	*curviflora*	21.71
48	*curviflora*	22.37
49	*curviflora*	21.30
50	*curviflora*	22.36
51	*curviflora*	22.78
52	*curviflora*	22.55
53	*curviflora*	22.93
54	*curviflora*	22.31
55	*curviflora*	20.49
56	*curviflora*	23.42
57	*curviflora*	20.86
58	*bolusiae*	3.43
59	*bolusiae*	3.54
60	*bolusiae*	3.56
61	*bolusiae*	3.31
62	*bolusiae*	3.37
63	*bolusiae*	3.16
64	*bolusiae*	3.37
65	*bolusiae*	3.29
66	*bolusiae*	2.89
67	*bolusiae*	3.07
68	*georgica*	9.43
69	*georgica*	8.75
70	*georgica*	9.25
71	*georgica*	8.62
72	*georgica*	9.37
73	*georgica*	8.59
74	*georgica*	8.43
75	*georgica*	8.70
76	*georgica*	9.13
77	*georgica*	9.35
78	*georgica*	7.84

79	*georgica*	8.57
80	*georgica*	8.48
81	*georgica*	9.02
82	*georgica*	8.84
85	*gracilis*	2.85
86	*gracilis*	3.26
87	*gracilis*	2.84
88	*gracilis*	2.78
89	*gracilis*	3.21
90	*gracilis*	3.22
91	*gracilis*	3.26
92	*gracilis*	3.08
93	*gracilis*	3.15
94	*gracilis*	3.25
95	*hirtiflora*	2.61
96	*hirtiflora*	2.97
97	*hirtiflora*	2.80
98	*hirtiflora*	2.82
99	*hirtiflora*	2.81
100	*hirtiflora*	2.92
101	*hirtiflora*	2.82
102	*hirtiflora*	2.78
103	*hirtiflora*	2.68
104	*hirtiflora*	2.54
105	*lateralis*	3.89
106	*lateralis*	3.56
107	*lateralis*	3.66
108	*lateralis*	3.97
109	*lateralis*	3.49
110	*lateralis*	3.76
111	*lateralis*	3.58
112	*lateralis*	3.68
113	*lateralis*	3.79
114	*lateralis*	3.54
115	*leucotrachela*	17.60
116	*leucotrachela*	18.41
117	*leucotrachela*	18.27
118	*leucotrachela*	17.52
119	*leucotrachela*	16.85
120	*leucotrachela*	17.82
121	*leucotrachela*	16.75
122	*leucotrachela*	18.06

123	*leucotrachela*	17.48
124	*leucotrachela*	19.73
125	*melanthera*	2.80
126	*melanthera*	2.72
127	*melanthera*	2.71
128	*melanthera*	2.69
129	*melanthera*	2.60
130	*melanthera*	2.91
131	*melanthera*	2.77
132	*melanthera*	2.87
133	*melanthera*	3.41
134	*melanthera*	2.77
135	*perspicua*	17.92
136	*perspicua*	18.70
137	*perspicua*	17.61
138	*perspicua*	18.57
139	*perspicua*	15.69
140	*perspicua*	17.29
141	*perspicua*	17.40
142	*perspicua*	18.62
143	*perspicua*	14.38
144	*perspicua*	17.61
145	*margaritacea*	2.90
146	*margaritacea*	2.91
147	*margaritacea*	4.18
148	*margaritacea*	4.15
149	*margaritacea*	3.74
150	*margaritacea*	2.88
151	*margaritacea*	3.99
152	*margaritacea*	4.08
153	*margaritacea*	2.80
154	*margaritacea*	4.12
155	*margaritacea*	3.60
156	*margaritacea*	2.72
157	*margaritacea*	2.84
158	*scoparia*	1.20
159	*scoparia*	1.29
160	*scoparia*	1.26
161	*scoparia*	1.27
162	*scoparia*	1.28
163	*scoparia*	1.26
164	*scoparia*	1.14

165	*scoparia*	1.29
166	*scoparia*	1.21
167	*scoparia*	1.15
168	*spiculifolia*	1.78
169	*spiculifolia*	1.66
170	*spiculifolia*	1.79
171	*spiculifolia*	1.77
172	*spiculifolia*	1.76
173	*spiculifolia*	1.76
174	*spiculifolia*	1.87
175	*spiculifolia*	1.73
176	*spiculifolia*	1.78
177	*spiculifolia*	1.74
178	*spiculifolia*	1.81
179	*spiculifolia*	1.83
180	*turgida*	2.30
181	*turgida*	2.31
182	*turgida*	2.11
183	*turgida*	2.03
184	*turgida*	2.25
185	*turgida*	2.03
186	*turgida*	2.21
187	*turgida*	2.20
188	*turgida*	2.18
189	*turgida*	2.09
190	*turgida*	2.14
191	*turgida*	2.25
192	*vagans*	1.90
193	*vagans*	2.01
194	*vagans*	1.99
195	*vagans*	2.17
196	*vagans*	1.96
197	*vagans*	1.98
198	*vagans*	1.99
199	*vagans*	1.82
200	*vagans*	1.86
201	*vagans*	1.97
202	*vagans*	1.99
204	*ventricosa*	13.83
205	*ventricosa*	13.93
206	*ventricosa*	13.78
207	*ventricosa*	13.74

208	*ventricosa*	13.94
209	*ventricosa*	13.73
210	*ventricosa*	14.27
211	*ventricosa*	13.83
212	*ventricosa*	13.46

Table S8. Classification of 114 individual flowers of diverse *Erica* species into the pollination syndromes, based on floral shape and size; proportions of assignation to each of the syndromes for each individual calculated based on predictions from 500 random forests of 1001 trees each.

flower number	*Erica* species	generalist	bird	long-proboscid fly	wind	sum
22	*blandifordia*	1	0	0	0	1
23	*blandifordia*	1	0	0	0	1
24	*blandifordia*	1	0	0	0	1
25	*blandifordia*	1	0	0	0	1
26	*blandifordia*	1	0	0	0	1
27	*blandifordia*	1	0	0	0	1
28	*blandifordia*	1	0	0	0	1
29	*blandifordia*	1	0	0	0	1
30	*blandifordia*	0	0	1	0	1
31	*blandifordia*	1	0	0	0	1
32	*blandifordia*	1	0	0	0	1
47	*curviflora*	0	1	0	0	1
48	*curviflora*	0	1	0	0	1
49	*curviflora*	0	1	0	0	1
50	*curviflora*	0	1	0	0	1
51	*curviflora*	0	1	0	0	1
52	*curviflora*	0	1	0	0	1
53	*curviflora*	0	1	0	0	1
54	*curviflora*	0	1	0	0	1
55	*curviflora*	0	1	0	0	1
56	*curviflora*	0	1	0	0	1
57	*curviflora*	0	1	0	0	1
58	*bolusiae*	1	0	0	0	1
59	*bolusiae*	1	0	0	0	1
60	*bolusiae*	1	0	0	0	1
61	*bolusiae*	1	0	0	0	1
62	*bolusiae*	1	0	0	0	1
63	*bolusiae*	1	0	0	0	1
64	*bolusiae*	1	0	0	0	1
65	*bolusiae*	1	0	0	0	1
66	*bolusiae*	1	0	0	0	1
67	*bolusiae*	1	0	0	0	1
68	*georgica*	0	1	0	0	1
69	*georgica*	0.016	0.294	0.69	0	1

70	*georgica*	0.007	0.993	0	0	1
71	*georgica*	0.061	0.939	0	0	1
72	*georgica*	0.049	0.951	0	0	1
73	*georgica*	0.239	0.759	0.002	0	1
74	*georgica*	0	1	0	0	1
75	*georgica*	0.338	0.662	0	0	1
76	*georgica*	0	1	0	0	1
77	*georgica*	0	1	0	0	1
78	*georgica*	1	0	0	0	1
79	*georgica*	0.994	0.006	0	0	1
80	*georgica*	1	0	0	0	1
81	*georgica*	0	1	0	0	1
82	*georgica*	0.018	0.982	0	0	1
105	*lateralis*	1	0	0	0	1
106	*lateralis*	1	0	0	0	1
107	*lateralis*	1	0	0	0	1
108	*lateralis*	1	0	0	0	1
109	*lateralis*	1	0	0	0	1
110	*lateralis*	1	0	0	0	1
111	*lateralis*	1	0	0	0	1
112	*lateralis*	1	0	0	0	1
113	*lateralis*	1	0	0	0	1
114	*lateralis*	1	0	0	0	1
115	*leucotrachela*	0	1	0	0	1
116	*leucotrachela*	0	1	0	0	1
117	*leucotrachela*	0	1	0	0	1
118	*leucotrachela*	0	1	0	0	1
119	*leucotrachela*	0	1	0	0	1
120	*leucotrachela*	0	1	0	0	1
121	*leucotrachela*	0	1	0	0	1
122	*leucotrachela*	0	1	0	0	1
123	*leucotrachela*	0	1	0	0	1
124	*leucotrachela*	0	1	0	0	1
125	*melanthera*	1	0	0	0	1
126	*melanthera*	1	0	0	0	1
127	*melanthera*	1	0	0	0	1
128	*melanthera*	1	0	0	0	1
129	*melanthera*	1	0	0	0	1
130	*melanthera*	1	0	0	0	1
131	*melanthera*	1	0	0	0	1
132	*melanthera*	1	0	0	0	1
133	*melanthera*	1	0	0	0	1

134	*melanthera*	1	0	0	0	1
145	*margaritacea*	1	0	0	0	1
146	*margaritacea*	1	0	0	0	1
147	*margaritacea*	1	0	0	0	1
148	*margaritacea*	1	0	0	0	1
149	*margaritacea*	1	0	0	0	1
150	*margaritacea*	1	0	0	0	1
151	*margaritacea*	1	0	0	0	1
152	*margaritacea*	1	0	0	0	1
153	*margaritacea*	1	0	0	0	1
154	*margaritacea*	1	0	0	0	1
155	*margaritacea*	1	0	0	0	1
156	*margaritacea*	1	0	0	0	1
157	*margaritacea*	1	0	0	0	1
168	*spiculifolia*	1	0	0	0	1
169	*spiculifolia*	1	0	0	0	1
170	*spiculifolia*	1	0	0	0	1
171	*spiculifolia*	1	0	0	0	1
172	*spiculifolia*	1	0	0	0	1
173	*spiculifolia*	1	0	0	0	1
174	*spiculifolia*	1	0	0	0	1
175	*spiculifolia*	1	0	0	0	1
176	*spiculifolia*	1	0	0	0	1
177	*spiculifolia*	1	0	0	0	1
178	*spiculifolia*	1	0	0	0	1
179	*spiculifolia*	1	0	0	0	1
180	*turgida*	1	0	0	0	1
181	*turgida*	1	0	0	0	1
182	*turgida*	1	0	0	0	1
183	*turgida*	1	0	0	0	1
184	*turgida*	1	0	0	0	1
185	*turgida*	1	0	0	0	1
186	*turgida*	1	0	0	0	1
187	*turgida*	1	0	0	0	1
188	*turgida*	1	0	0	0	1
189	*turgida*	1	0	0	0	1
190	*turgida*	1	0	0	0	1
191	*turgida*	1	0	0	0	1

Table S9. Support values evolutionary models of floral shape evolution. Support for BM, OU, and EB models for the evolution of highly-dimensional whole floral shape in *Erica*; preferred model (lowest GIC value) in bold.).

	GIC	Log-likelihood	parameters
BM	-13276	6766	-
**OU**	**-13325**	**6793**	**Alpha = 2.57**
EB	-13274	6766	r = 0

Table S10. Summary of the preferred models of evolution for seven phenotypic trait variables (PC1-5 of floral shape, centroid size, and integration) under the pollination-syndrome regime. Parameter estimates are reported as a mean ± standard error. Models of quantitative trait evolution with their parameters, indicating for each model whether θ (the optimum trait value), σ^2 (the intensity of random fluctuation in the evolutionary trajectory), and α (the selective pull toward the optimal value) are modelled with one global parameter (BM_1 and OU_1) or four parameters (OU_M) that are specific for each pollination syndrome.

Shape and size variables	Model	θ				σ^2	α
		Generalist	Bird	LPF	Wind		
Centroid size	OUM	10,36±1,56	49,95±2,91	31,92±6,63	-2,51±7,13	95.79	1.63
PC1 (39% variance)	OUM	0,09±0,03	-0,35±0,06	-0,72±0,25	0,50±0,15	1.36E-02	0.68
PC2 (22% variance)	OU1	3.49E-03±3.47E-02				1.23E-02	0.32
PC3 (11% variance)	BM1	5.35E-02±6.19E-02				7.54E-04	-
PC4 (7% variance)	BM1	2.85E-02±5.66E-02				6.32E-04	-
PC5 (6% variance)	OU1	1.41E-03±1.78E-02				3.71E-03	0.35
Integration	OU1	0,23±0,02				1.15E-03	0.18
